# Commentary: CU06-1004 inhibits the progression of chronic colitis and colitis-associated colorectal cancer by suppressing inflammation

**DOI:** 10.3389/fphar.2025.1750070

**Published:** 2026-01-05

**Authors:** Wentao Guo, Yun Wang, Danqin Fang, Zhihui Zeng

**Affiliations:** 1 Shenzhen TCM Anorectal Hospital (Futian), Shenzhen, China; 2 Shenzhen Traditional Chinese Medicine Hospital, Shenzhen, China

**Keywords:** CU06-1004, colitis-associated colorectal cancer (CAC), vascular stabilization, inflammation, therapeutic targeting

## Introduction

In the rapidly evolving landscape of inflammatory bowel disease (IBD) research, the study by Kim et al. (2025) offers a timely exploration into the therapeutic potential of CU06-1004, a small molecule with vascular-stabilizing properties, for mitigating chronic colitis and its progression to colitis-associated colorectal cancer (CAC). This original research adeptly bridges the gap between acute inflammation models and chronic disease settings, presenting compelling evidence that CU06-1004 suppresses key inflammatory pathways and oncogenic signaling in murine models. The findings ignite enthusiasm for vascular-targeted strategies as a novel approach to interrupting the inflammation-cancer axis, which is particularly relevant given the rising global burden of colorectal cancer linked to IBD and the well-documented limitations of existing therapies (Neurath, 2017). However, while the study provides valuable insights, it also invites critical reflection on mechanistic depth and translational applicability, themes that will be unpacked in this commentary to guide future research endeavors. The compelling narrative of CU06-1004 as a dual-action agent against inflammation and carcinogenesis sets the stage for a nuanced discussion on optimizing its path from bench to bedside.

## Mechanistic evaluation and pathway interrogation

Kim et al. (2025) convincingly demonstrate that CU06-1004 alleviates chronic colitis and CAC by reducing immune cell infiltration and pro-inflammatory cytokine levels, with downstream suppression of β-catenin and c-Myc expression. The study excels in using well-established models like DSS-induced chronic colitis and AOM/DSS-induced CAC, which recapitulate human disease progression reasonably well. The integration of histological, molecular, and systemic analyses strengthens the claim that CU06-1004 reinforces endothelial barrier function, thereby curtailing leukocyte recruitment through adhesion molecule inhibition—a mechanism that aligns with emerging interests in targeting vascular inflammation in IBD (Neurath, 2017), similar to approaches in cancer therapy where PFKFB3 inhibition promotes vessel normalization (Cantelmo et al., 2016). Nonetheless, the mechanistic elucidation remains somewhat superficial; for instance, the link between reduced inflammation and β-catenin downregulation is inferred rather than directly proven, leaving room for ambiguity regarding whether this effect is primary or secondary to attenuated inflammation. To solidify this causal relationship, future studies could employ techniques such as spatial transcriptomics (Marx, 2021) to precisely map the co-localization of endothelial stabilization, inflammatory cytokine reduction, and β-catenin activation within the tumor microenvironment. This would clarify whether β-catenin downregulation is a direct consequence of reduced endothelial permeability and subsequent diminution of inflammatory cues on epithelial cells, or a secondary effect. Furthermore, utilizing endothelial-specific conditional knockout mice(e.g., VE-cadherin Cre) for key signaling molecules would be indispensable to conclusively demonstrate the vasculature-centric nature of CU06-1004’s action, distinguishing it from potential direct effects on epithelial or immune cells. This gap is noteworthy because β-catenin signaling is a cornerstone of colorectal carcinogenesis, and its modulation by inflammatory cues is complex, involving crosstalk with pathways like NF-κB and STAT3, which are only briefly addressed. A more granular investigation, such as using endothelial-specific knockout models, could clarify if CU06-1004s effects are vasculature-centric or involve epithelial cell autonomous mechanisms. Moreover, while the study highlights reduced cytokine levels, it does not delve into the temporal dynamics of these changes, which could reveal critical windows for intervention. The absence of data on potential off-target effects or interactions with other barrier components, such as the microbiome, further limits the mechanistic narrative. Thus, while the findings are promising, they underscore the need for deeper pathway validation to establish CU06-1004’s specificity and avoid overinterpretation of its anti-tumorigenic role.

## Model limitations and translational considerations

The reliance on murine models in Kim et al. (2025) is both a strength and a weakness, as these systems provide a controlled environment but may not fully emulate human IBD heterogeneity. The DSS and AOM/DSS models are widely accepted for their ability to induce inflammation-driven carcinogenesis, yet they primarily mimic ulcerative colitis-like conditions, overlooking the nuances of Crohn’s disease or the genetic diversity seen in patients. For example, the study uses male ICR mice, which may not account for sex-specific differences in IBD prevalence or response to therapy, a limitation that becomes apparent when contrasting the homogeneous experimental conditions with the complex patient populations described in clinical guidelines (Torres et al., 2020). Additionally, the preventive administration of CU06-1004 at disease onset contrasts with clinical scenarios where treatment often begins after chronic inflammation is established; this design choice raises questions about the compound’s efficacy in reversal or maintenance therapy, which are key considerations in modern treatment paradigms aiming for mucosal healing (Torres et al., 2020). To directly address this translational gap, a critical next step would be to evaluate CU06-1004 in a therapeutic intervention model, where treatment is initiated after the establishment of chronic colitis or even upon the appearance of early dysplastic lesions. This would more accurately simulate the clinical setting of treating patients with longstanding IBD. Moreover, assessing the combinatorial efficacyof CU06-1004 with standard-of-care biologics (e.g., anti-TNF agents) or immune checkpoint inhibitors is paramount, as supported by recent evidence on combination therapy in IBD (Dai et al., 2023). Given its role in vascular normalization and modulation of the immune microenvironment, CU06-1004 may synergize with these therapies to enhance drug delivery and overcome resistance, a hypothesis that aligns with the push for multi-target strategies in complex diseases like IBD. The study commendably assesses systemic inflammation via serum cytokines but misses opportunities to evaluate long-term safety or combinatorial effects with existing IBD therapies, such as biologics, which are paramount for translational relevance. Furthermore, while the reduction in tumor number and size is statistically significant, the clinical implications for human CAC prevention remain speculative without dose-response studies or pharmacokinetic data. These limitations highlight the importance of complementing animal work with human organoid cultures or patient-derived xenografts to better predict efficacy. As such, future research should prioritize models that incorporate human immune components or environmental factors, like diet, to enhance the predictive value of findings and bridge the gap between preclinical promise and clinical application.

## Discussion

The study by Kim et al. (2025) makes a substantial contribution to the field by positioning CU06-1004 as a multifaceted inhibitor of inflammation-driven colorectal cancer, yet it also reveals areas for refinement. The critical assessment herein suggests that while the vascular-focused mechanism is innovative, the evidence would benefit from enhanced mechanistic rigor and broader model diversity to solidify its translational potential. The critical assessment herein charts a clear path for future validation. To bridge the gap between compelling preclinical findings and clinical application, efforts should be prioritized in three areas (1) employing advanced molecular mapping tools (e.g., spatial transcriptomics): to delineate the cellular crosstalk underpinning CU06-1004’s effect on the inflammation-cancer axis; (2) validating the vasculature-specific mechanism using genetically engineered models; and (3) rigorously testing its therapeutic and combinatorial potential in clinically relevant settings. Such a multi-faceted approach will be crucial to harness the full potential of vascular-targeted therapies for CAC prevention. For instance, incorporating spatial transcriptomics could map the cellular interactions in the tumor microenvironment, and investigating CU06-1004 in combination with immunotherapies might unveil synergistic effects, an approach aligned with the push for multi-target strategies in complex diseases like IBD (Neurath, 2017). Importantly, the study’s strengths—such as the comprehensive *in vivo* validation—provide a solid foundation for these future directions. In conclusion, this commentary advocates for a balanced approach that builds on Kim et al.’s findings while addressing their limitations, ultimately advancing CU06-1004 as a candidate for preventing CAC in high-risk IBD populations. By fostering interdisciplinary collaboration and rigorous validation, researchers can harness the full potential of vascular-targeted therapies to combat the daunting challenge of inflammation-associated cancers.
